# Heavy metal exposure and risk of all-cause and cardiovascular mortality in population with cardiovascular-kidney-metabolic syndrome stage 0–3: a cohort study

**DOI:** 10.1265/ehpm.26-00065

**Published:** 2026-07-03

**Authors:** Yiyang Liu, Fujian Li, Ying Huang, Jiansheng Cai, You Li

**Affiliations:** 1 School of Public Health, Guilin Medical University, Guilin 541199, China; 2 Guangxi Hospital Division of the First Affiliated Hospital, Sun Yat-sen University, Nanning 530021, China; 3 Sub-Center of Key Laboratory of Environmental Pollution and Integrative Omics (Education Department of Guangxi Zhuang Autonomous Region), Lingshan Hospital of Guilin Medical University, Lingshan 535400, PR China

**Keywords:** CKM syndrome, Blood metals, Mortality, Cardiovascular mortality, NHANES

## Abstract

**Background:**

Evidence on the association between blood metal exposure and mortality among adults with cardiovascular–kidney–metabolic (CKM) syndrome stages 0–3 remains limited. We examined the associations of blood lead (Pb), cadmium (Cd), mercury (Hg), selenium (Se), and manganese (Mn) with all-cause and cardiovascular mortality in this population.

**Methods:**

We analyzed data from 4,394 adults with CKM stages 0–3 from the National Health and Nutrition Examination Survey (NHANES) 2011–2018 linked to mortality records. Blood metal concentrations were measured using inductively coupled plasma mass spectrometry. Weighted Cox proportional hazards models, restricted cubic spline (RCS) analyses, and weighted quantile sum (WQS) regression were used to assess associations of individual and mixed metal exposures with mortality outcomes.

**Results:**

In the fully adjusted model, elevated blood Cd levels were associated with a higher risk of all-cause mortality (HR = 1.71, 95% CI: 1.26–2.33). In contrast, blood Se showed an inverse association with all-cause mortality, with lower risks observed in Q2 (HR = 0.61, 95% CI: 0.41–0.90) and Q4 (HR = 0.59, 95% CI: 0.38–0.93). RCS analyses showed nonlinear associations of Cd and Se with all-cause mortality. Cd exhibited an inverted U-shaped pattern, whereas Se showed an L-shaped inverse association. WQS regression suggested a positive association between mixed metal exposure and all-cause mortality (HR = 1.16, 95% CI: 0.99–1.34; P = 0.046). Associations with cardiovascular mortality were weaker and less consistent.

**Conclusions:**

Among U.S. adults with stage 0–3 cardiovascular–kidney–metabolic syndrome, elevated blood cadmium levels and reduced blood selenium levels were potentially associated with a higher risk of all-cause mortality.

**Supplementary information:**

The online version contains supplementary material available at https://doi.org/10.1265/ehpm.26-00065.

## Introduction

CKM syndrome is a multisystem disorder arising from the interplay among obesity, diabetes, chronic kidney disease (CKD), and cardiovascular disease (CVD). It is associated with premature mortality, substantial morbidity, and considerable healthcare burden, and has become a major global public health concern [1]. Although these conditions were previously considered distinct entities, accumulating evidence suggests that they share common pathophysiological mechanisms and are closely interconnected [2]. Current data indicate that nearly 90% of U.S. adults are classified as CKM stage 1 or higher, and approximately 15% have progressed to advanced stages [3]. Given that CVD, diabetes, and CKD are leading causes of death, and that their coexistence may further amplify mortality risk, identifying modifiable determinants among individuals within the CKM spectrum is of substantial clinical and public health importance [4]. In response to growing recognition of the interconnected cardiometabolic, renal, and cardiovascular continuum, the American Heart Association (AHA) has defined CKM as a continuum of disease states associated with metabolic dysfunction and/or excess adiposity [5]. CKM stages 0–3 represent a prevention-oriented continuum, ranging from the absence of apparent CKM risk factors to metabolic risk factors, CKD, or subclinical CVD before the onset of clinically manifest CVD [12]. The AHA scientific statement emphasizes primordial and primary prevention among individuals with CKM stages 0–3 before progression to CKM stage 4, which is characterized by established clinical CVD [6]. Therefore, adults with CKM stages 0–3 represent an important target population for early risk assessment and preventive intervention.

Although CKM management increasingly emphasizes integrated care, most previous studies have focused on traditional risk factors, whereas environmental exposures remain relatively understudied [7]. Metal exposure has been increasingly recognized as a potential contributor to cardiovascular dysfunction, renal injury, and metabolic disturbances [8–10]. Because CKM involves overlapping metabolic, renal, and cardiovascular abnormalities, individuals with CKM stages 0–3 may be particularly vulnerable to the adverse effects of metal exposure. However, evidence regarding the association between blood metal exposure and mortality in this population remains limited. Therefore, using data from NHANES 2011–2018, this study investigated the associations of individual and mixed blood concentrations of Pb, Cd, Hg, Se, and Mn with all-cause and cardiovascular mortality among U.S. adults with CKM stages 0–3.

## Methods

### Participants

**Fig. 1:**
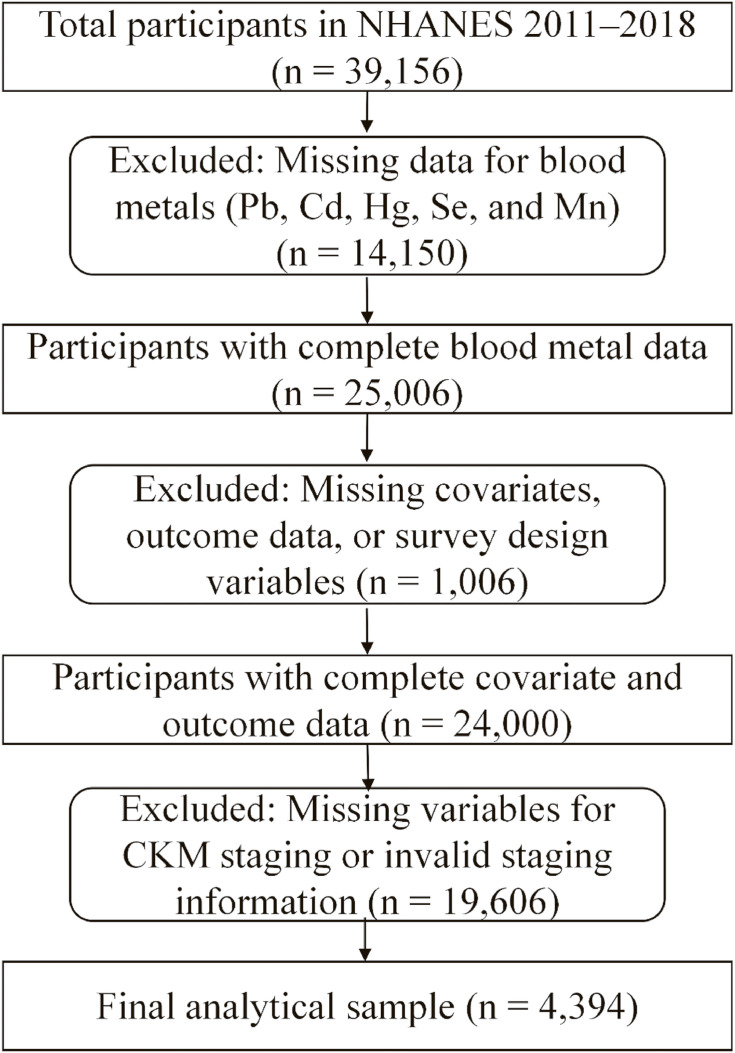
Flowchart of participant selection for the analysis of NHANES 2011–2018 data.

Data were obtained from four consecutive cycles of NHANES 2011–2018. NHANES, conducted by the National Center for Health Statistics (NCHS) under the Centers for Disease Control and Prevention (CDC), is a nationally representative, multistage probability survey of the U.S. civilian noninstitutionalized population. The survey is designed to assess the prevalence of major diseases and related risk factors and to provide data for nutrition and health policy development. The NHANES protocol was approved by the NCHS Research Ethics Review Board, and all adult participants provided written informed consent. A total of 39,156 participants from NHANES 2011–2018 were initially considered. Participants were excluded if they had missing data on any of the five blood metals Pb, Cd, Hg, Se, or Mn n = 14,150, leaving 25,006 participants. We further excluded individuals with missing demographic variables age, sex, educational attainment, or race/ethnicity; lifestyle factors alcohol consumption, physical activity, smoking status, or poverty–income ratio PIR; outcome variables all-cause mortality, cardiovascular mortality, or follow-up time; or complex survey design variables strata, primary sampling units, or survey weights n = 1,006, leaving 24,000 participants. Participants with missing variables required for CKM staging were also excluded. These variables included waist circumference, blood pressure, fasting glucose, lipid profiles, and estimated glomerular filtration rate (eGFR). Participants classified as CKM stage 4 were excluded because this study focused on prevention before clinically manifest CVD. CKM stage 4 was defined as established clinical CVD, including coronary heart disease, heart failure, myocardial infarction, or stroke, or advanced kidney disease/end-stage renal disease, including eGFR <15 mL/min/1.73 m^2^ or dialysis. This restriction was intended to reduce confounding related to disease severity, medication use, lifestyle modification, and secondary prevention after clinical CVD. After all exclusions, 4,394 participants with CKM stages 0–3 were included in the final analysis. The participant selection process is shown in Fig. 1.

### Determination of CKM

Participants were classified according to the CKM staging framework proposed by the AHA. Briefly, CKM stage 0 was defined as the absence of metabolic, kidney, or cardiovascular risk factors. Stage 1 included excess or dysfunctional adiposity or impaired glucose regulation. Stage 2 was characterized by metabolic risk factors or CKD. Stage 3 indicated subclinical CVD in the presence of CKM risk factors, whereas stage 4 represented clinically manifest CVD in the context of CKM. Further details are provided in Supplement materials 1.

Several physiological indicators relevant to CKM classification were used, including eGFR and 10-year CVD risk. eGFR was calculated using the 2021 race- and ethnicity-independent Chronic Kidney Disease Epidemiology Collaboration (CKD-EPI) equation, which incorporates age, sex, and serum creatinine [13, 14]. In addition, the study employed the Prevent Heart Disease equation (PREVENT equation) to estimate 10-year absolute CVD risk. This predictive model integrates multiple variables, including age, sex, systolic blood pressure, use of antihypertensive medication, total cholesterol, high-density lipoprotein cholesterol, use of statin therapy, diabetes status, smoking status, eGFR, and body mass index (BMI). All continuous variables within the model were assigned standardized preset cutoff values; if a variable exceeded its predefined range, truncation was applied to bring the value into the valid analytic interval. The PREVENT equation was used to estimate 10-year absolute CVD risk. This model incorporates age, sex, systolic blood pressure, antihypertensive medication use, total cholesterol, high-density lipoprotein cholesterol, statin use, diabetes status, smoking status, eGFR, and body mass index BMI. Continuous variables were truncated to prespecified valid ranges when values exceeded the analytic limits of the model.

### Collection of blood metal measurements

Blood metal concentrations were measured according to official NHANES 2011–2018 laboratory protocols and relevant literature [15]. Briefly, whole blood samples were diluted at a 1:10 ratio with ultrapure nitric acid HNO_3_ to reduce matrix interference and protect the sample introduction system. Concentrations of Pb, Cd, Hg, Mn, and Se were quantified using inductively coupled plasma mass spectrometry (ICP-MS), such as the Agilent 7700x platform. Because of its high sensitivity and ability to simultaneously quantify multiple elements, ICP-MS is widely used in large-scale epidemiological studies of metal exposure [16]. All procedures followed NHANES laboratory quality-control protocols. Calibration was performed using National Institute of Standards and Technology (NIST)-certified standard reference materials, including SRM 955c and SRM 966b. Method blanks, laboratory blanks, and duplicate samples one duplicate per 20 samples were included in each analytical batch. Intra-batch and inter-batch coefficients of variation were maintained at ≤5% and ≤8%, respectively, ensuring measurement accuracy and reproducibility. For concentrations below the lower limit of detection (LLOD), values were imputed as LLOD divided by √ 2, a conventional approach in epidemiological studies that preserves information on low-level exposure while reducing bias from left-censored data [17].

### Determination of variables

Covariates were selected based on prior CKM and metal exposure studies [11, 18]. As this study draws its data from the NHANES database, the race/ethnicity categories employed herein are fully aligned with the NHANES classification, conforming to epidemiological research standards for the U.S. population. Educational attainment is recognized as an important confounder of health outcomes in CKM populations [1, 3]. The classification used—ranging from less than high school to an associate degree or higher—matches that of the present study, enabling assessment of the influence of socioeconomic status on health outcomes. Consistent with established research [19, 20], we applied the NHANES standard cut-off for low income, defined as a poverty–income ratio (PIR) < 1.3, a criterion widely adopted for income stratification in U.S. population health research. Regarding health behavior information, the definitions of smoking and alcohol consumption were derived from previous CKM and metal exposure studies [11, 21], which provided a direct reference and employed identical definitions, allowing for effective control of lifestyle-related confounding. The standardized approach to physical activity assessment, based on self-reported intensity levels as utilized in previous CKM and mortality risk evaluations [4, 9], which is consistent with the measurement approach adopted in this study. Accordingly, the covariates in this study included variables in the following categories. The demographic and socioeconomic variables comprised age (continuous), sex (male, female), race/ethnicity (non-Hispanic White, non-Hispanic Black, Mexican American, other race/ethnicities), educational attainment (less than 9th grade; 9th–11th grade, including 12th grade without diploma; high school graduate/GED or equivalent; some college or associate degree; college graduate or above), and household income (low income: PIR < 1.3; high income: PIR ≥ 1.3). The definitions of hypertension, diabetes mellitus, and metabolic syndrome were based on NHANES questionnaire data, physical examination results, laboratory measurements, and established clinical criteria. Hypertension was defined as a self-reported physician diagnosis, current use of antihypertensive medication, or measured blood pressure ≥140/90 mmHg. Diabetes mellitus was defined as a self-reported physician diagnosis, current use of glucose-lowering medication, fasting plasma glucose ≥7.0 mmol/L, or non-fasting blood glucose ≥11.1 mmol/L. Metabolic syndrome was defined in accordance with the 2009 Joint Interim Statement as the presence of at least three of the following five criteria: abdominal obesity, elevated triglycerides, reduced high-density lipoprotein cholesterol, elevated blood pressure, and elevated fasting glucose. Smoking status was categorized as never smoker or ever smoker. Never smokers were defined as individuals who had smoked fewer than 100 cigarettes in their lifetime, whereas ever smokers included former and current smokers who had smoked at least 100 cigarettes or reported current smoking. Alcohol consumption was classified as consumer or non-consumer. Consumers were defined as individuals who had consumed at least 12 alcoholic drinks in the past year or had a history of consuming at least 12 drinks in any single year. Physical activity was assessed based on self-reported participation in vigorous and moderate-intensity activities.

### Mortality ascertainment and outcome definition

The primary outcome was all-cause mortality, and the secondary outcome was cardiovascular mortality. Mortality status was ascertained through probabilistic linkage of NHANES records to the National Death Index (NDI) maintained by the NCHS, with follow-up through December 31, 2019. Causes of death were classified using the International Classification of Diseases, Tenth Revision ICD-10. All-cause mortality included death from any cause. Cardiovascular mortality included deaths due to heart disease ICD-10 codes I00–I09, I11, I13, and I20–I51 and cerebrovascular disease ICD-10 codes I60–I69.

### Statistical analysis

All analyses accounted for the complex NHANES survey design by incorporating survey weights, strata, and primary sampling units (PSUs), consistent with official NHANES analytic guidelines [22]. Categorical variables are presented as frequencies and weighted percentages. Continuous variables with non-normal distributions are presented as medians with interquartile ranges. Between-group differences were assessed using design-adjusted chi-square tests for categorical variables and survey-weighted nonparametric tests for continuous variables.

Blood metal concentrations were categorized into quartiles Q1–Q4, with Q1 as the reference group. Metal concentrations were also natural log-transformed to improve distributional properties. Associations between blood metal exposure and all-cause or cardiovascular mortality were evaluated using Cox proportional hazards regression. Two models were fitted: an unadjusted model and a fully adjusted model controlling for age, sex, educational attainment, race/ethnicity, alcohol consumption, physical activity, smoking status, PIR, and total energy intake. Hazard ratios (HRs) and 95% confidence intervals (Cis) were estimated for each exposure quartile relative to Q1. Survival differences across exposure quartiles were evaluated using log-rank tests.

Dose–response relationships were assessed using restricted cubic spline RCS models with degrees of freedom df = 4, with knots placed at the 10th, 50th, and 90th percentiles of each metal distribution. WQS regression was used to evaluate the joint association of the five-metal mixture with mortality outcomes and to identify the principal contributors to the mixture effect. WQS regression is a commonly used method in environmental epidemiology for assessing health effects of correlated chemical mixtures [23]. To evaluate potential interactions among metals, all pairwise multiplicative interaction terms among Pb, Cd, Hg, Se, and Mn were examined. Metal concentrations were natural log-transformed and standardized before generating interaction terms. For each metal pair, a survey-weighted Cox proportional hazards model was fitted, including the two corresponding metal main effects, their product term, and covariates. Models were adjusted for age, sex, educational attainment, race/ethnicity, alcohol consumption, physical activity, smoking status, and poverty income ratio. P values for interaction were reported, and false discovery rate correction was additionally applied to account for multiple comparisons. All analyses were conducted using R version 4.5.1. A two-sided P value < 0.05 was considered statistically significant.

## Results

### Baseline characteristics of study participants

As shown in Table 1, 4,394 participants from NHANES 2011–2018 were included in the final analysis, representing U.S. adults with CKM stages 0–3 after application of survey weights. The median age was approximately 56 years, and hypertension and diabetes were common at baseline. The median blood concentrations of Pb, Cd, Hg, Se, and Mn were approximately 1.10 µg/dL, 0.34 µg/dL, 0.82 µg/dL, 193 µg/dL, and 9.4 µg/dL, respectively. Participants were classified according to all-cause and cardiovascular mortality status; for cardiovascular mortality, those who remained alive or died from non-cardiovascular causes were included in the non-event group.

**Table 1:** Basic Information and All-Cause and Cardiovascular Mortality at baseline

**Variable**	**Alive**	**All-cause death**	**P-value^1^**	**Alive/Non-CVD death**	**Cardiovascular death**	**P-value^2^**
**Demographics**						
Age, years	54 (42–65)	74 (61–80)	<0.001	55 (42–66)	75 (67–80)	<0.001
Male, n (%)	1953 (48%)	183 (61%)	<0.001	2081 (48%)	55 (65%)	0.003
**Race/Ethnicity, n (%)**			<0.001			0.027
- Non-Hispanic White	1617 (39%)	181 (61%)		1752 (41%)	46 (54%)	
- Non-Hispanic Black	889 (22%)	56 (19%)		924 (21%)	21 (25%)	
- Others^3^	1589 (39%)	62 (20%)		1633 (38%)	18 (21%)	
**Socioeconomic & Lifestyle**						
High School Grad or above, n (%)	3240 (79%)	203 (68%)	<0.001	3240 (79%)	203 (68%)	0.002
Poverty income ratio (<1.3), n (%)	1187 (29%)*	111 (37%)*	0.002	1220 (29%)	28 (33%)	0.6
Current or Former Smoker, n (%)	1834 (45%)	185 (62%)	<0.001	1975 (46%)	44 (52%)	0.3
Physically Inactive, n (%)	2374 (58%)	213 (71%)	<0.001	2522 (59%)	65 (76%)	0.008
Alcohol Consumption (Yes), n (%)	2613 (64%)	212 (71%)	0.013	2771 (64%)	54 (64%)	0.9
**Clinical Conditions**						
CKM Stage 3, n (%)	466 (11%)	105 (35%)	<0.001	532 (12%)	39 (46%)	<0.001
Hypertension, n (%)	2614 (64%)	252 (84%)	<0.001	2793 (65%)	73 (86%)	<0.001
Diabetes (Type 2), n (%)	1003 (24%)	124 (41%)	<0.001	1087 (25%)	40 (47%)	<0.001
Metabolic Syndrome, n (%)	2188 (53%)	186 (62%)	0.003	2315 (54%)	59 (69%)	0.004
Moderate/Severe CKD^4^, n (%)	689 (16.5%)	144 (48.4%)	<0.001	788 (18%)	45 (53%)	0.027
**Blood Metals (Exposure)**						
Pb, µg/dL	1.08 (0.7–1.7)	1.58 (1.1–2.5)	<0.001	1.11 (0.7–1.7)	1.58 (1.1–2.5)	<0.001
Cd, µg/dL	0.32 (0.2–0.6)	0.51 (0.3–0.8)	<0.001	0.33 (0.2–0.6)	0.45 (0.3–0.8)	<0.001
Hg, µg/dL	0.82 (0.4–1.6)	0.67 (0.4–1.3)	0.008	0.81 (0.4–1.6)	0.74 (0.4–1.7)	0.5
Se, µg/dL	193 (178–209)	187 (173–205)	<0.001	193 (178–208)	189 (175–208)	0.2
Mn, µg/dL	9.4 (7.5–11.7)	8.3 (6.6–10.7)	<0.001	9.3 (7.5–11.7)	8.2 (5.9–11.3)	

Notes: Continuous variables are presented as median (interquartile range, IQR); categorical variables are presented as ***n*** (weighted %).

^1^P-values compare the Alive and All-cause death groups.

^2^P-values compare the Alive/Non-CVD death and Cardiovascular death groups.

^3^“Others” includes Mexican American, Other Hispanic, and Other Races.

^4^Moderate/Severe CKD corresponds to CKD stages 3, 4, and 5.

*Percentages for PIR were recalculated based on the complement of your original data to show the high-risk (lower income) group.

Compared with survivors, participants who died from any cause were older, more likely to be male, had lower educational attainment, less favorable lifestyle profiles, and a greater burden of cardiometabolic comorbidities. They also had higher blood Pb and Cd concentrations but lower Hg, Se, and Mn concentrations. Similar patterns were observed for cardiovascular mortality: participants who died from cardiovascular causes were older, more likely to be male, and had higher prevalences of physical inactivity and cardiometabolic comorbidities than those without cardiovascular death. Blood Pb and Cd concentrations were also higher in the cardiovascular death group, whereas differences in Hg and Se were not statistically significant.

### Associations and dose-response relationships between blood metal concentrations and mortality

**Fig. 2:**
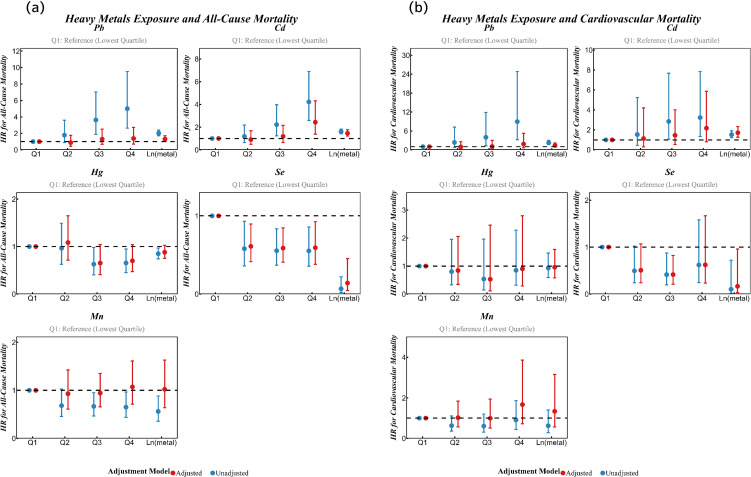
Summary of Forest Plots for All-Cause and Cardiovascular Mortality Figure a shows HRs for all-cause mortality across quartiles of blood concentrations of Pb, Cd, Hg, Se, and Mn. Figure b shows HRs for cardiovascular mortality for the same metal exposure categories. Error bars represent 95% confidence intervals for both unadjusted (blue) and adjusted (red) models. The reference group (Q1) corresponds to the Q1 of exposure.

Kaplan–Meier survival curves shown in Supplement materials 2 indicated significant differences in survival probability across quartiles of blood metal concentrations log-rank P < 0.01 for all metals. As shown in Fig. 2, fully adjusted Cox proportional hazards models showed that higher blood Cd concentrations were associated with increased all-cause mortality (HR = 1.71, 95%CI: 1.26–2.33). In contrast, blood Se concentrations were inversely associated with all-cause mortality. Compared with Q1, the risk of all-cause mortality was lower in Q2 (HR = 0.61, 95%CI: 0.41–0.90), Q3 (HR = 0.59, 95%CI: 0.41–0.85), and Q4 (HR = 0.59, 95%CI: 0.38–0.93). For cardiovascular mortality, blood Se showed a similar inverse association. Compared with Q1, the risk of cardiovascular mortality was lower in Q2 (HR = 0.51, 95%CI: 0.24–1.07), Q3 (HR = 0.42, 95%CI: 0.21–0.82), and Q4 (HR = 0.65, 95%CI: 0.34–1.25).

**Fig. 3:**
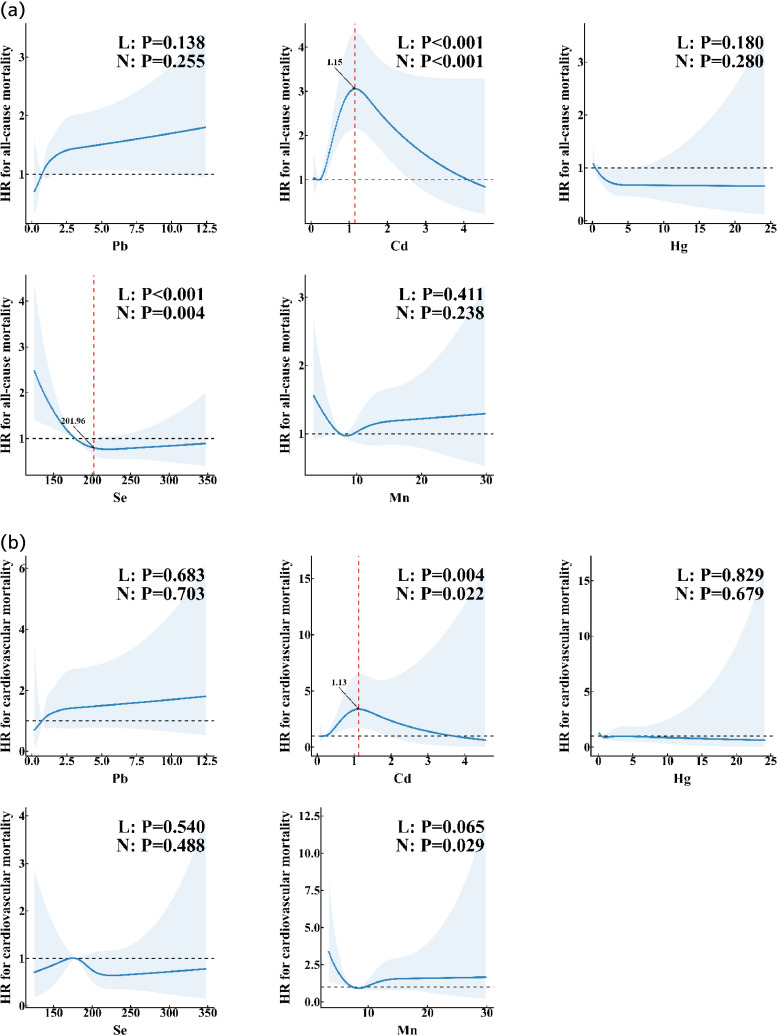
Summary of RCS for All-Cause and Cardiovascular Mortality Dose–response relationships between blood concentrations of Pb, Cd, Hg, Se, and Mn and the risks of all-cause mortality (a) and cardiovascular mortality (b). Solid lines represent hazard ratios estimated from RCS models, and shaded areas indicate 95% confidence intervals. “L” and “N” denote P values for linear and nonlinear trends, respectively. Red vertical lines mark the identified turning points for Cd and Se, where the direction or slope of the dose–response relationship changes notably.

As illustrated in Fig. 3, RCS analyses further supported nonlinear dose–response relationships of Cd and Se with all-cause mortality. Cd showed a significant nonlinear association with all-cause mortality P for overall < 0.001; P for nonlinearity < 0.001. The estimated risk increased with increasing blood Cd concentrations and peaked at approximately 1.2 µg/dL, after which the curve declined. Se also demonstrated a significant nonlinear inverse association with all-cause mortality, showing an L-shaped pattern P for overall < 0.001; P for nonlinearity = 0.004. As blood Se concentrations increased, the estimated risk decreased, with the risk reduction becoming less pronounced after approximately 202 µg/dL.

For cardiovascular mortality, RCS analyses suggested nonlinear associations for Cd and Mn. Restricted cubic spline analysis showed a nonlinear association between Cd and cardiovascular mortality, with the estimated risk increasing at lower exposure levels and declining after a potential inflection point at approximately 1.1 µg/dL.

### Mixture exposures of blood metals and risks of mortality

**Fig. 4:**
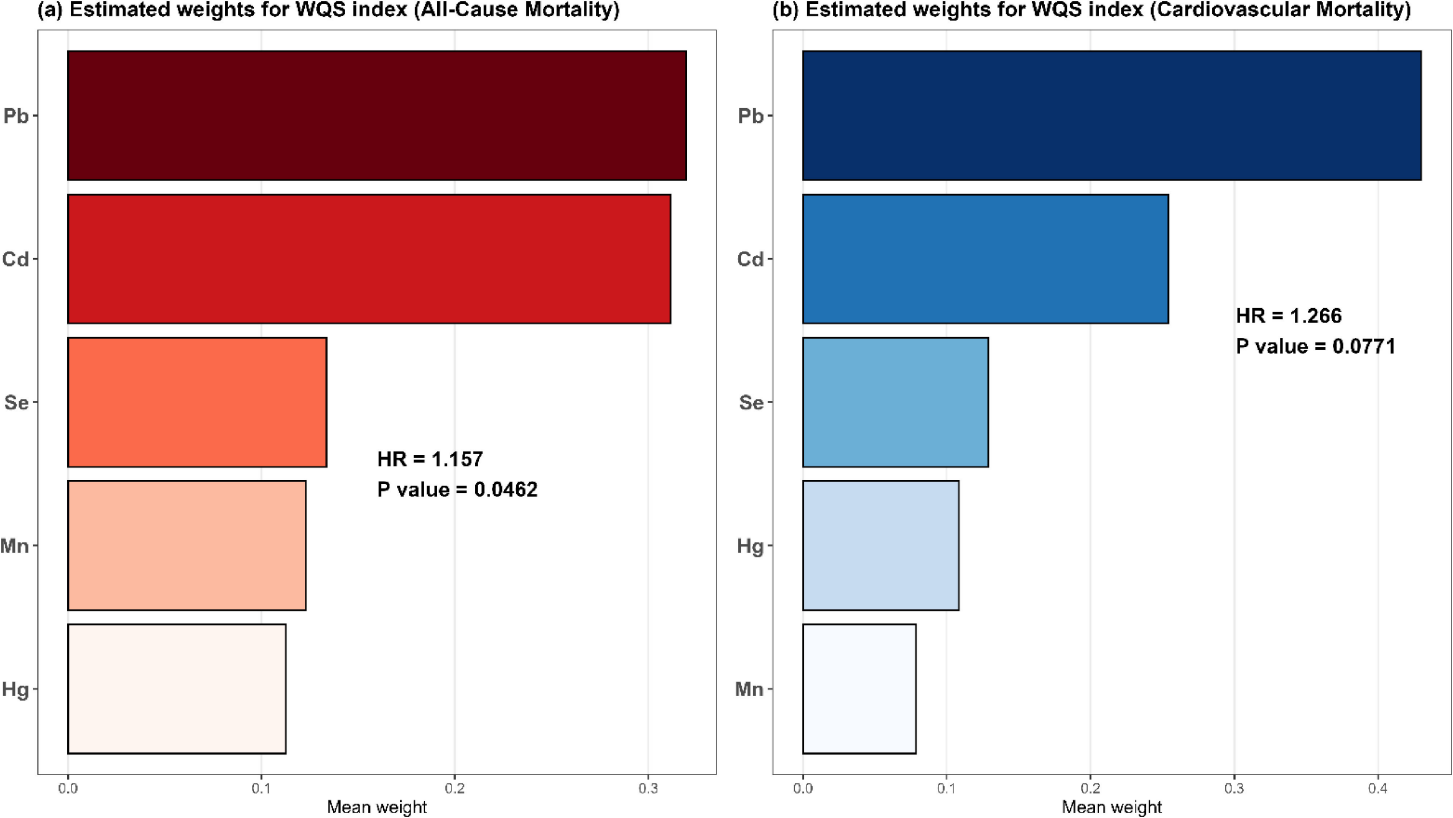
Summary of Weight Comparison and WQS Index Hazard Ratios for All-Cause and Cardiovascular Mortality The WQS analysis results for heavy metals in relation to mortality. The panel shows the weight percentages of individual metals—Cd, Hg, Mn, Pb, and Se—for all-cause mortality (a) and CVD mortality (b) models.

As shown in Fig. 4a, WQS regression indicated a positive association between mixed metal exposure and all-cause mortality. Each one-unit increase in the WQS index was associated with a 15.7% higher risk of all-cause mortality (HR = 1.16; 95% CI: 0.99–1.34; P = 0.046). The relative weights for all-cause mortality were ranked as Pb, Cd, Se, Mn, and Hg in descending order.

For cardiovascular mortality Fig. 4b, the metal mixture showed a positive but non-significant association with risk (HR = 1.27; P = 0.077), with weights ranked as Pb, Cd, Se, Hg, and Mn in descending order. Supplement materials 3 showed pairwise interaction analyses, no statistically significant multiplicative interactions were observed among blood Pb, Cd, Hg, Se, and Mn in relation to all-cause or cardiovascular mortality. The interaction between Pb and Cd was not significantly associated with all-cause or cardiovascular mortality. No other pairwise metal interaction terms were statistically significant, and the results remained non-significant after false discovery rate correction.

## Discussion

In this nationally representative cohort of U.S. adults with CKM stages 0–3, we identified several notable associations between blood metal concentrations and mortality. First, blood Cd was independently associated with increased all-cause mortality, whereas Pb showed no statistically significant independent association after multivariable adjustment. Second, Se showed an L-shaped inverse association with all-cause mortality, suggesting higher risk at low concentrations and potential benefit at moderate levels. Third, mixed metal exposure was positively associated with all-cause mortality in WQS regression, with Pb contributing the largest weight to the mixture effect. These findings extend existing evidence by characterizing individual and combined metal–mortality associations specifically in adults within the prevention-oriented CKM spectrum.

Cd was significantly and positively associated with both all-cause and cardiovascular mortality in participants with CKM, which is consistent with findings from multiple previous epidemiological studies. In populations with CKD, metabolic syndrome, and the general population, Cd exposure has been linked to renal function decline, metabolic dysregulation, and increased mortality risk [24–26]. Its nephrotoxicity and role in promoting cardiovascular events have been well documented across diverse cohorts [27]. Specifically, large CKM cohort studies spanning different disease stages have reported that higher Cd exposure is associated with faster declines in eGFR, with late-stage CKM patients experiencing substantially greater cardiovascular mortality risk than those at earlier stages, even after adjustment for demographic and clinical covariates [24]. Experimental evidence further supports these observational findings: in rat models of Cd-induced renal injury, marked alterations in renal m6A methylation profiles have been observed, characterized by increased methylation and expression of oxidative stress- and apoptosis-related genes, leading to extensive tubular epithelial cell apoptosis and providing direct mechanistic evidence of Cd nephrotoxicity [28]. Beyond its well-established nephrotoxicity, Cd also exerts direct detrimental effects on the cardiovascular system and contributes to increased mortality. A comprehensive review and meta-analysis identified Cd as a key contributor to atherosclerosis, with multiple underlying mechanisms, including oxidative stress, inflammation, endothelial dysfunction, enhanced lipid synthesis, adhesion molecule upregulation, prostaglandin imbalance, and altered glycosaminoglycan synthesis [29]. Similarly, studies specifically examining low-dose Cd exposure have highlighted its role in promoting atherosclerosis through multiple signaling pathways and through endothelial dysfunction and vascular tissue damage [30]. For instance, one study showed that environmental Cd exposure reprograms macrophage inflammatory states, driving excessive chemokine release and sustained ACKR1-dependent macrophage–endothelial interactions. This cascade promotes oxidative stress, inflammation, and endothelial dysfunction within atherosclerotic lesions, thereby accelerating atherosclerosis progression [31]. Supporting this mechanism, animal studies have shown altered plasma lipid profiles, hepatic lipid accumulation, and dysregulation of genes involved in cholesterol uptake and efflux following Cd exposure, while clinical evidence has linked plasma miR-30d-5p levels with Cd exposure and stroke risk [32]. The present study suggests that Cd-related vascular, cardiac, and nephrotoxic effects may jointly contribute to increased cardiovascular mortality. In addition, mouse co-exposure studies involving polystyrene microplastics and Cd have demonstrated synergistic renal oxidative stress, with Cd-induced renal injury increasing by more than 40%, suggesting that combined environmental exposures may partly explain inconsistencies across previous studies [33]. Such inconsistent findings are more likely in populations with occupational exposure, older age, or complex pollutant mixtures, where confounding is more difficult to fully address. Mechanistically, Cd induces mitochondrial reactive oxygen species, activates the renin–angiotensin system, and promotes tubular apoptosis and endothelial dysfunction through inflammatory pathways, collectively contributing to metabolic dysregulation and cardiorenal injury [28]. Previous studies have consistently reported adverse renal and cardiovascular effects associated with Pb exposure. Even at low concentrations, blood Pb has been significantly associated with CKD incidence and renal function decline [34]. In the general population and among individuals with metabolic syndrome, Pb concentrations show monotonic positive relationships with all-cause and cardiovascular mortality [25]. Overall, effect heterogeneity across studies appears limited, although in populations with severe comorbidities, Pb-related effects may be partially obscured by competing disease processes. Epidemiological follow-up in large community cohorts has shown that blood Pb levels below 50 µg/L, the traditional safety threshold, remain significantly associated with renal function decline; each 10 µg/L increase in blood Pb has been linked to an approximately 3.2 mL/min reduction in creatinine clearance, with stronger associations observed in CKM populations [34]. Renal biopsy studies further indicate that individuals co-exposed to low levels of Pb, Cd, and Hg exhibit more pronounced mesangial proliferation and tubular atrophy, with Pb exposure positively correlated with histopathological injury scores [35]. Animal studies have demonstrated that combined exposure to Pb and Cd additively activates the renin–angiotensin–aldosterone system, resulting in substantially greater endothelial dysfunction than single-metal exposure, confirming their synergistic toxicity [21]. At the mechanistic level, Pb toxicity involves activation of the renin–angiotensin–aldosterone system, oxidative stress induction, and endothelial dysfunction, with amplified effects in the presence of Cd and other metals [36]. In the present study, blood Pb did not exhibit a statistically significant independent dose–response association with mortality in RCS analyses; however, it contributed prominently to the overall mixture effect identified in WQS models. This apparent discrepancy likely reflects the differing objectives of these modeling approaches: RCS evaluates independent exposure–response relationships, whereas WQS emphasizes the relative contribution of correlated components within a mixture. Given the strong correlation between Pb and Cd, Pb in this context may function primarily as a co-exposure indicator within the metal mixture rather than as an independent dominant risk factor, a pattern that has also been reported in previous mixture exposure studies.

Se demonstrated an L-shaped inverse association with all-cause mortality in our CKM cohort, which is consistent with findings from numerous studies in populations with CVD, CKD, and older adults. The physiological basis for this nonlinear pattern lies in Se’s dual nature as both an essential micronutrient and a potentially toxic element at supraphysiological concentrations. Its beneficial effects are mainly mediated through selenoproteins, which are indispensable for antioxidant defense, including glutathione peroxidases and thioredoxin reductases, as well as for thyroid hormone metabolism and immune function [37]. Se may also antagonize the toxicity of heavy metals such as Cd and Hg [38, 39]. Given selenium’s well-documented cardiorenal benefits, the lack of a significant association with cardiovascular mortality in our CKM population merits consideration. This paradox may reflect several factors: in patients with advanced CVD, selenium’s protective effects may be masked by severe risk burden or irreversible tissue damage; a single selenium measurement may not adequately capture long-term exposure status; and residual confounding or a unique optimal selenium range in this high-risk group may obscure true associations. The significant association between Se and all-cause mortality likely reflects selenium’s broader protective roles rather than a specific cardiovascular effect in this population. Nevertheless, inconsistent findings have been reported in some subgroups, particularly unstratified metabolic syndrome cohorts and mixed-metal exposure models. In unselected populations, selenium’s apparent benefits may be weakened by exposure misclassification or heterogeneity in cardiometabolic risk profiles [16, 40]. In mixed-exposure settings, Se’s effects may be modified by interactions with other trace elements, or may reach a plateau under conditions of high Cd burden, where selenium-dependent detoxification pathways become saturated [19, 41]. Evidence from the CHNS further supports this L-shaped relationship, showing that moderate serum Se concentrations are associated with the lowest CKD risk, whereas higher levels confer no additional benefit [42]. Likewise, NHANES analyses have confirmed the protective role of Se, reporting an inverse association between dietary Se intake and CKM prevalence, an association that was especially pronounced among individuals with elevated Cd exposure, highlighting a potential beneficial role of Se in mitigating heavy metal-related risks in high-risk subgroups [19]. Mechanistically, experimental studies suggest that Se protects against renal injury by suppressing NF-κB-mediated inflammation and modulating aquaporin-1 expression, thereby reducing oxidative stress, preserving tubular function, and attenuating cardiorenal deterioration [39].

No significant associations between Hg or Mn exposure and mortality risk were observed, consistent with most prior studies. Hg has not shown clear associations with all-cause or cardiovascular mortality in large cohorts, including the UK Biobank and mining populations [26, 41], nor has it emerged as a major risk factor in diabetes or metabolic syndrome cohorts [25, 43]. Mn, an essential trace element, has not been associated with increased mortality risk at typical environmental exposure levels, with toxicity primarily confined to high-dose exposure [40]. Potential explanations for these null findings include exposure levels below toxicity thresholds, the dominance of more toxic metals such as Cd and Pb within exposure mixtures, and residual confounding by lifestyle factors [41].

This study has several strengths. First, we focused on adults with CKM stages 0–3, a prevention-oriented population aligned with the latest AHA framework. This design facilitates evaluation of environmental risk factors before clinically manifest CVD develops. Second, the use of a nationally representative NHANES sample with standardized laboratory assays and mortality linkage enhances the reliability and generalizability of the findings. Third, our analytical approach combined RCS models to characterize nonlinear associations and WQS regression to evaluate mixed metal exposure, providing a more comprehensive assessment than single-pollutant models alone.

Several limitations should also be acknowledged. First, baseline blood metal concentrations may not fully represent long-term exposure. For some metals, particularly Pb, blood levels may reflect recent exposure or mobilization from internal stores rather than cumulative lifetime exposure. Second, although multiple confounders were adjusted for, residual confounding remains possible. Dietary factors, such as seafood intake, may influence both metal concentrations and cardiovascular outcomes. Third, the association between the WQS index and cardiovascular mortality was not statistically significant, possibly because of the limited number of cardiovascular deaths and reduced statistical power for this secondary outcome. Finally, the observational design precludes causal inference. Future longitudinal studies incorporating repeated exposure assessments and metallomic profiling are needed to clarify causal pathways linking metal exposure to mortality in CKM populations.

## Conclusion

Among U.S. adults with CKM stages 0–3, elevated blood Cd levels and reduced blood Se levels were potentially associated with a higher risk of all-cause mortality. Further prospective and mechanistic studies are needed to confirm these associations and elucidate the underlying biological pathways.

## Abbreviations

CKM: cardiovascular–kidney–metabolic syndrome
CKD: chronic kidney disease
CVD: cardiovascular disease
Pb: lead
Cd: cadmium
Hg: mercury
Se: selenium
Mn: manganese
CDC: Centers for Disease Control and Prevention
AHA: American Heart Association
eGFR: estimated glomerular filtration rate
NF-κB: nuclear factor kappa-B
CKD-EPI: Chronic Kidney Disease Epidemiology Collaboration
PIR: poverty–income ratio
BMI: body mass index
ICP–MS: inductively coupled plasma–mass spectrometry
NIST: National Institute of Standards and Technology
LLOD: lower limit of detection
DR1IKCA: day 1 dietary recall energy intake (kcal)
DR2TKCAL: day 2 dietary recall energy intake (kcal)
NHANES: National Health and Nutrition Examination Survey
NCHS: National Center for Health Statistics
PSUs: primary sampling units
ROS: reactive oxygen species
AQP-1: aquaporin-1

## Supplementary information

**Additional file 1:** Supplementary Detailed Definition and Staging of Cardiovascular–Kidney–Metabolic (CKM) Risk.

**Additional file 2:** **Figure S1.** Kaplan–Meier Survival Curves for All-Cause Mortality by Quartiles of Blood Metal Concentrations.

**Additional file 3:** **Supplementary Table 1.** Pairwise interaction analyses of blood metals in relation to mortality.

## Declarations

### Availability of data and material

The data that support the findings of this study are available from the corresponding author upon reasonable request. Requests to access the datasets should be directed to Yiyang Liu.
