# Supplementary material for: Heavy metal exposure and risk of all-cause and cardiovascular mortality in population with cardiovascular-kidney-metabolic syndrome stage 0–3: a cohort study
Source: Environ Health Prev Med. 2026 Jul 3;31:45. doi: 10.1265/ehpm.26-00065 (PMC13366184; doi:10.1265/ehpm.26-00065)
Supplement: Supplementary file 1 — Additional file 1: Supplementary Detailed Definition and Staging of Cardiovascular–Kidney–Metabolic (CKM) Risk. [file ehpm-31-045-s001.pdf]

Online Resource 1. Supplementary Detailed Definition and Staging of Cardiovascular–Kidney–Metabolic (CKM) Risk

Article title: Heavy metal exposure and risk of all-cause and cardiovascular mortality in population with cardiovascular-kidney-metabolic syndrome stage 0–3: a cohort study

Author information

Yiyang Liu[1] · Fujian Li[2] · Ying Huang[1] · Jiansheng Cai[1, 3]· You Li[1]

[1]School of Public Health, Guilin Medical University, Guilin 541199, China
[2]Guangxi Hospital Division of the First Affiliated Hospital, Sun Yat- sen University, Nanning 530021, China

[3]Sub-Center of Key Laboratory of Environmental Pollution and Integrative Omics (Education Department of Guangxi Zhuang Autonomous Region), Lingshan Hospital of Guilin Medical University, Lingshan 535400, PR China

Yiyang Liu and Fujian Li equally contributed to this work.

🖂 Corresponding authors:

You Li (liyou121300@163.com)
Jiansheng Cai (15007714226@163.com)

Description:

Detailed Definition and Staging of Cardiovascular–Kidney–Metabolic (CKM) Risk

I. Definition of CKM Risk. Cardiovascular–kidney–metabolic (CKM) risk is an integrated stratification framework that encompasses chronic kidney disease (CKD), cardiovascular disease (CVD), and metabolic risk factors (e.g., obesity, dysglycemia, dyslipidemia, and hypertension). It aims to comprehensively assess combined risk across the cardiorenal–metabolic spectrum and to inform precision prevention and control strategies (based on the AHA's most recent CKM staging paradigm and Kittelson et al., 2024).

II. Core Basis for Staging. CKM staging in this study follows the core logic of "metabolic risk factors–degree of cardiorenal injury–clinical outcomes," determined by the integration of three domains:

Metabolic risk factors: obesity (general or central), impaired glucose regulation, diabetes, dyslipidemia, hypertension.

Cardiorenal structural/functional injury: CKD stage, subclinical cardiovascular damage

(e.g., atherosclerosis, left ventricular hypertrophy).

Clinical endpoints: established cardiovascular disease (e.g., coronary artery disease, stroke, heart failure).

III. Detailed Diagnostic Criteria by Stage.

Stage 0: No-Risk Stage

Core criteria: no metabolic risk factors and no evidence of cardiorenal structural/functional injury.

Specific determination:

Metabolic domain: no general obesity (BMI < 25 kg/m²); no central obesity (waist circumference < 90 cm in men, < 85 cm in women); fasting plasma glucose (FPG) < 6.1 mmol/L (no impaired fasting glucose); HbA1c < 5.7%; normal lipids (total cholesterol < 5.2 mmol/L; triglycerides < 1.7 mmol/L; HDL-C ⩾ 1.04 mmol/L in men and ⩾ 1.3 mmol/L in women); normal blood pressure (SBP < 130 mmHg and DBP < 85 mmHg, without antihypertensive therapy).

Cardiorenal domain: eGFR ⩾ 90 mL/min/1.73 m² (no CKD), no subclinical cardiovascular damage (e.g., normal carotid intima–media thickness, no LV hypertrophy), no history of CVD.

Stage 1: Incipient Metabolic Risk

Core criteria: isolated obesity or impaired glucose regulation, without cardiorenal structural/functional injury.

Specific determination (any of the following):

Isolated obesity: BMI ⩾ 25 kg/m² (or central obesity) with otherwise normal glycemia, lipids, and blood pressure and no cardiorenal injury.

Impaired glucose regulation: FPG 6.1–7.0 mmol/L (impaired fasting glucose), 2- hour post-load glucose 7.8–11.1 mmol/L (impaired glucose tolerance), or HbA1c 5.7%–6.4%, with no obesity (or obesity but no other metabolic abnormalities) and no cardiorenal injury.

Stage 2: Metabolic Abnormality and/or Cardiorenal Injury

Core criteria: on the basis of Stage 1, presence of at least one additional metabolic abnormality, or CKD, without established CVD.

Specific determination (any of the following):

Added metabolic abnormality: existing obesity and/or impaired glucose regulation plus any of the following—hypertension (SBP ⩾ 130 mmHg, DBP ⩾ 85 mmHg, or antihypertensive use), dyslipidemia (per the lipid criteria above), or diagnosed type 2 diabetes (FPG ⩾ 7.0 mmol/L or HbA1c ⩾ 6.5%).

Chronic kidney disease (CKD): eGFR 60–89 mL/min/1.73 m^2 (stage 1–2 CKD) or albumin- to- creatinine ratio (ACR) 30–300 mg/g (microalbuminuria), with or without concurrent metabolic abnormalities, but no established CVD.

Stage 3: Subclinical CKM Syndrome

Core criteria: presence of subclinical cardiovascular damage together with metabolic dysfunction or definite CKD.

Specific determination:

Subclinical cardiovascular damage: evidence of atherosclerosis (carotid intima–media thickness ⩾ 1.0 mm or plaque) and/or left ventricular hypertrophy (echocardiographic LV mass index ⩾ 115 g/m^2 in men, ⩾ 95 g/m^2 in women).

Concomitant conditions: coexisting metabolic dysfunction (e.g., type 2 diabetes, mixed dyslipidemia, resistant hypertension) or definite CKD (eGFR 30–59 mL/min/1.73 m^2; stage 3 CKD).

Exclusion: no established CVD (e.g., coronary artery disease, myocardial infarction, stroke, heart failure).

Stage 4: Clinical CKM Disease

Core criteria: overt cardiovascular disease with concomitant metabolic and/or renal injury.

Specific determination:

Established cardiovascular disease: diagnosed coronary artery disease, acute myocardial infarction, ischemic/hemorrhagic stroke, heart failure, or peripheral artery disease.

Concomitant injury: metabolic injury (e.g., uncontrolled diabetes, dyslipidemia) and/or renal injury (eGFR < 30 mL/min/1.73 m^2, representing stage 4–5 CKD, or ACR ⩾ 300 mg/g, representing macroalbuminuria).
