# Supplementary material for: Heavy metal exposure and risk of all-cause and cardiovascular mortality in population with cardiovascular-kidney-metabolic syndrome stage 0–3: a cohort study
Source: Environ Health Prev Med. 2026 Jul 3;31:45. doi: 10.1265/ehpm.26-00065 (PMC13366184; doi:10.1265/ehpm.26-00065)
Supplement: Supplementary file 2 — Additional file 2: Figure S1. Kaplan–Meier Survival Curves for All-Cause Mortality by Quartiles of Blood Metal Concentrations. [file ehpm-31-045-s002.pdf]

Online Resource 2.

Figure S1. Kaplan–Meier Survival Curves for All-Cause Mortality by Quartiles of Blood Metal Concentrations

Article title: Heavy metal exposure and risk of all-cause and cardiovascular mortality in population with cardiovascular-kidney-metabolic syndrome stage 0–3: a cohort study

Author information

Yiyang Liu[1] · Fujian Li[2] · Ying Huang[1] · Jiansheng Cai[1, 3]· You Li[1]

[1]School of Public Health, Guilin Medical University, Guilin 541199, China
[2]Guangxi Hospital Division of the First Affiliated Hospital, Sun Yat- sen University, Nanning 530021, China

[3]Sub-Center of Key Laboratory of Environmental Pollution and Integrative Omics (Education Department of Guangxi Zhuang Autonomous Region), Lingshan Hospital of Guilin Medical University, Lingshan 535400, PR China

Yiyang Liu and Fujian Li equally contributed to this work.

🖂 Corresponding authors:

You Li (liyou121300@163.com)
Jiansheng Cai (15007714226@163.com)

Description

Kaplan–Meier survival curves for all-cause mortality are shown for quartiles (Q1–Q4) of blood concentrations of lead (Pb), cadmium (Cd), mercury (Hg), selenium (Se), and manganese (Mn) among 4,394 adults with stage 0–3 cardiovascular–kidney–metabolic (CKM) syndrome from the NHANES 2011–2018 dataset.

Blood metal concentrations were determined via inductively coupled plasma–mass spectrometry and categorized into quartiles. Survival differences were evaluated using log-rank tests.

Main findings:

Pb: Higher quartiles were associated with a significantly lower survival probability (log-rank $P < 0.001$).

Cd: Higher quartiles had a strong and consistent negative association with survival (log-rank $P < 0.001$).

Hg: A significant survival difference was observed across quartiles (log-rank P = 0.008).

Se: Higher quartiles were associated with a better survival, indicating a protective pattern (log-rank P < 0.001).

Mn: Significant survival differences were found among quartiles (log-rank P < 0.001).

Abbreviations: Pb = lead; Cd = cadmium; Hg = mercury; Se = selenium; Mn = manganese; Q = quartile.

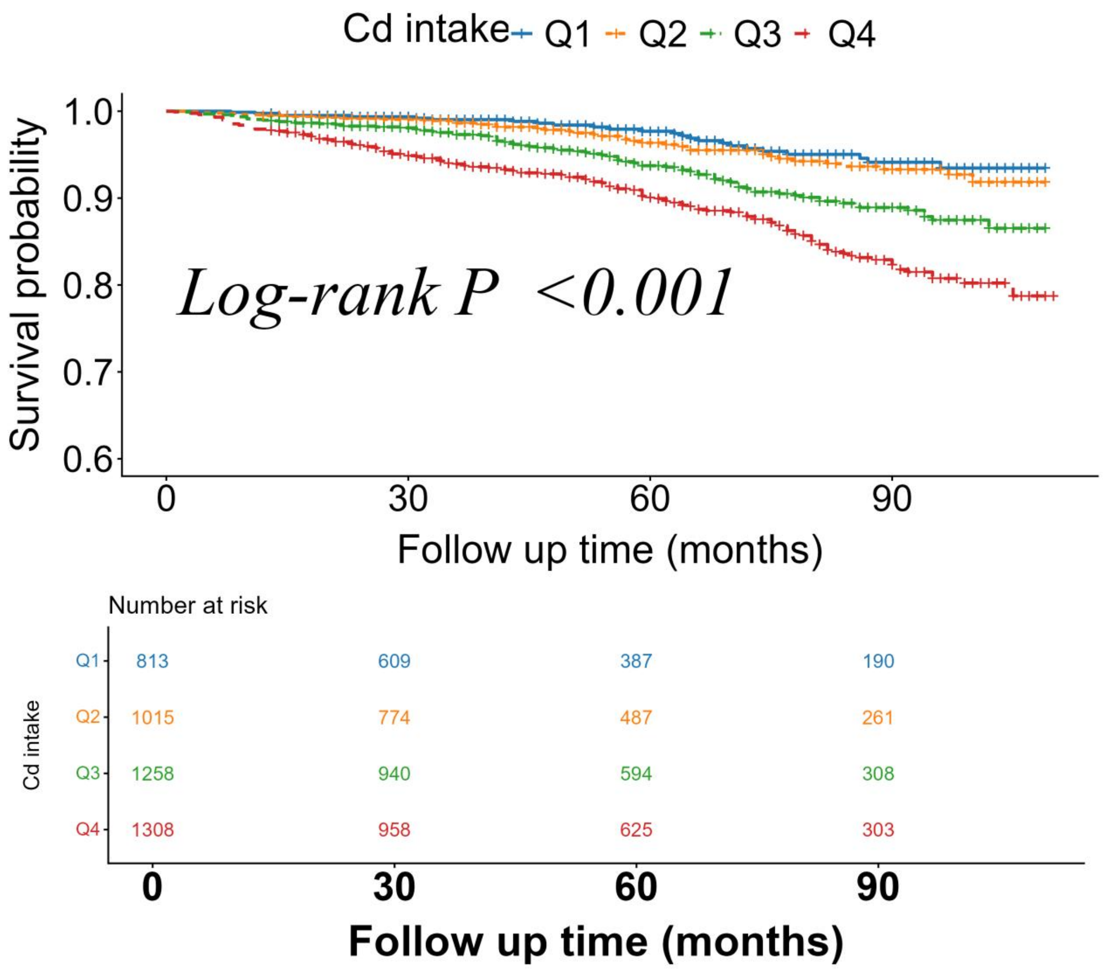

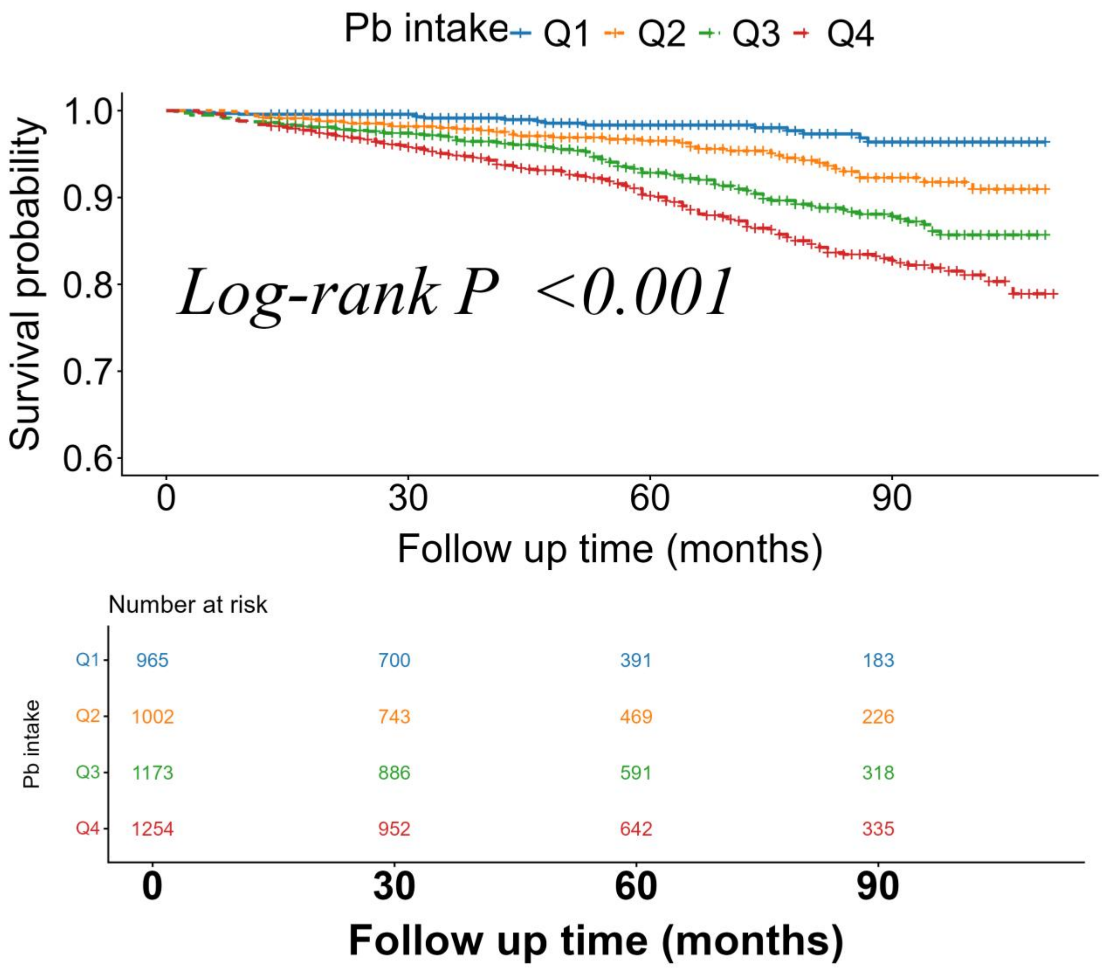

Pb intake
Q1
Q2
Q3
Q4
Survival probability
1.0
0.9
0.8
0.7
0.6
Log-rank P <0.001
0
30
60
90
Follow up time (months)
Number at risk
Pb intake
Q1 965 700 391 183
Q2 1002 743 469 226
Q3 1173 886 591 318
Q4 1254 952 642 335
0
30
60
90
Follow up time (months)

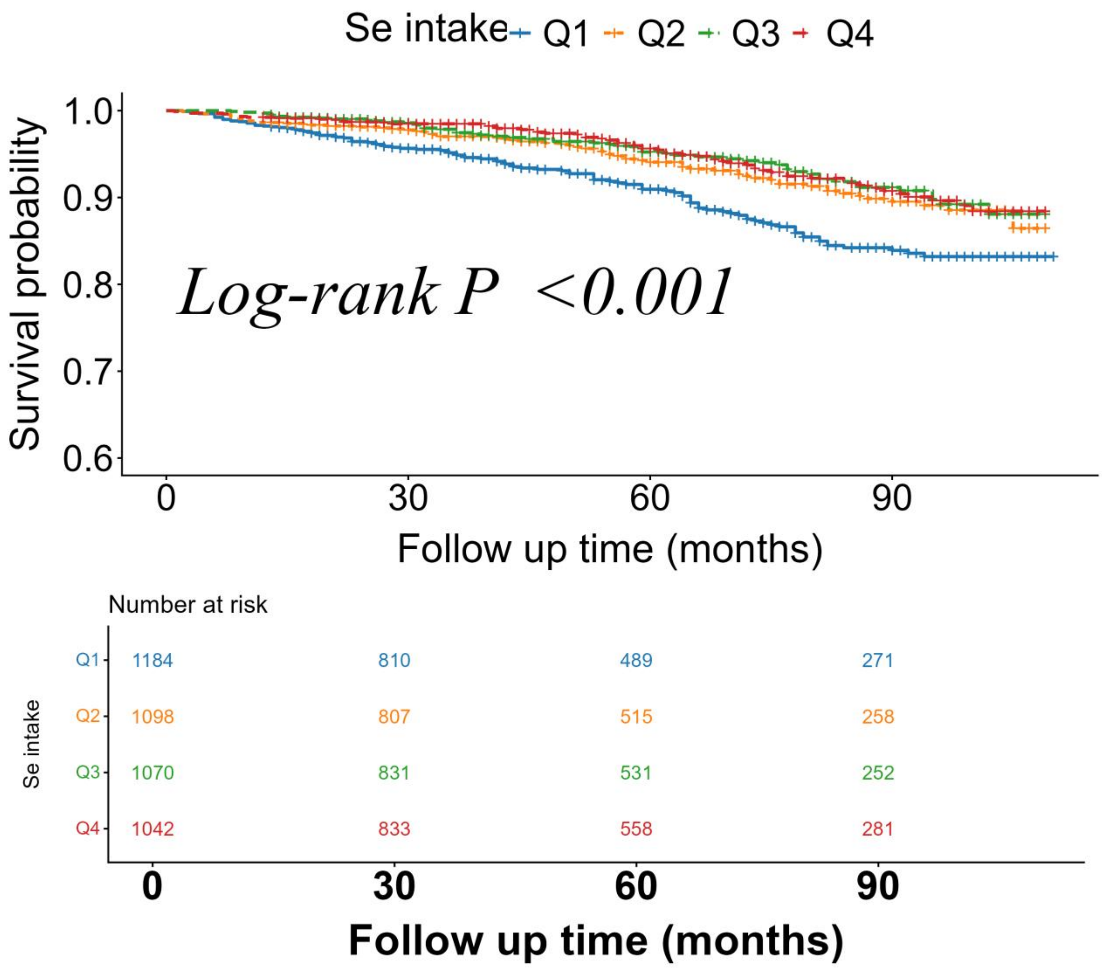

Se intake
Q1
Q2
Q3
Q4
Survival probability
1.0
0.9
0.8
0.7
0.6
Log-rank P <0.001
0
30
60
90
Follow up time (months)
Number at risk
Se intake
Q1 1184 810 489 271
Q2 1098 807 515 258
Q3 1070 831 531 252
Q4 1042 833 558 281
0
30
60
90
Follow up time (months)

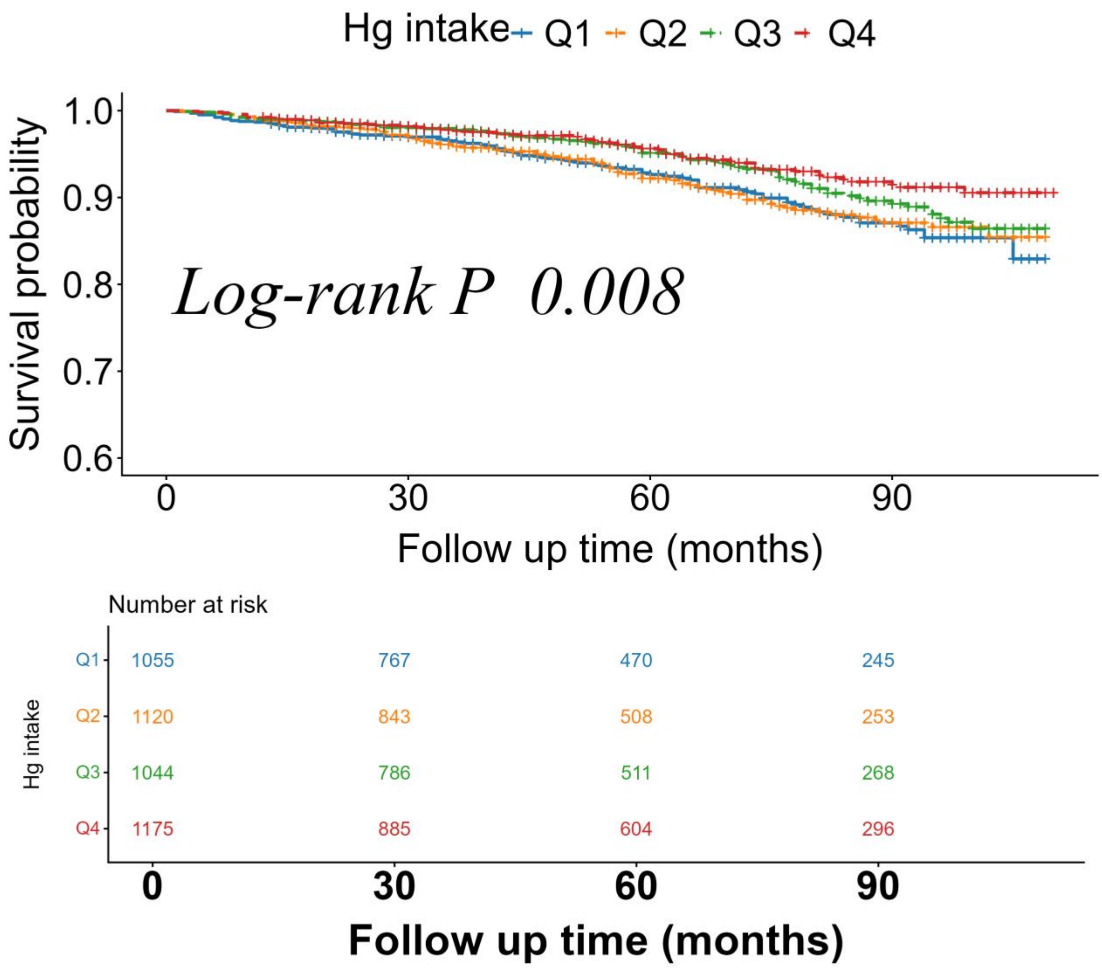

Hg intake
Q1
Q2
Q3
Q4
Survival probability
1.0
0.9
0.8
0.7
0.6
Log-rank P 0.008
0
30
60
90
Follow up time (months)
Number at risk
Hg intake
Q1 1055 767 470 245
Q2 1120 843 508 253
Q3 1044 786 511 268
Q4 1175 885 604 296
0
30
60
90
Follow up time (months)

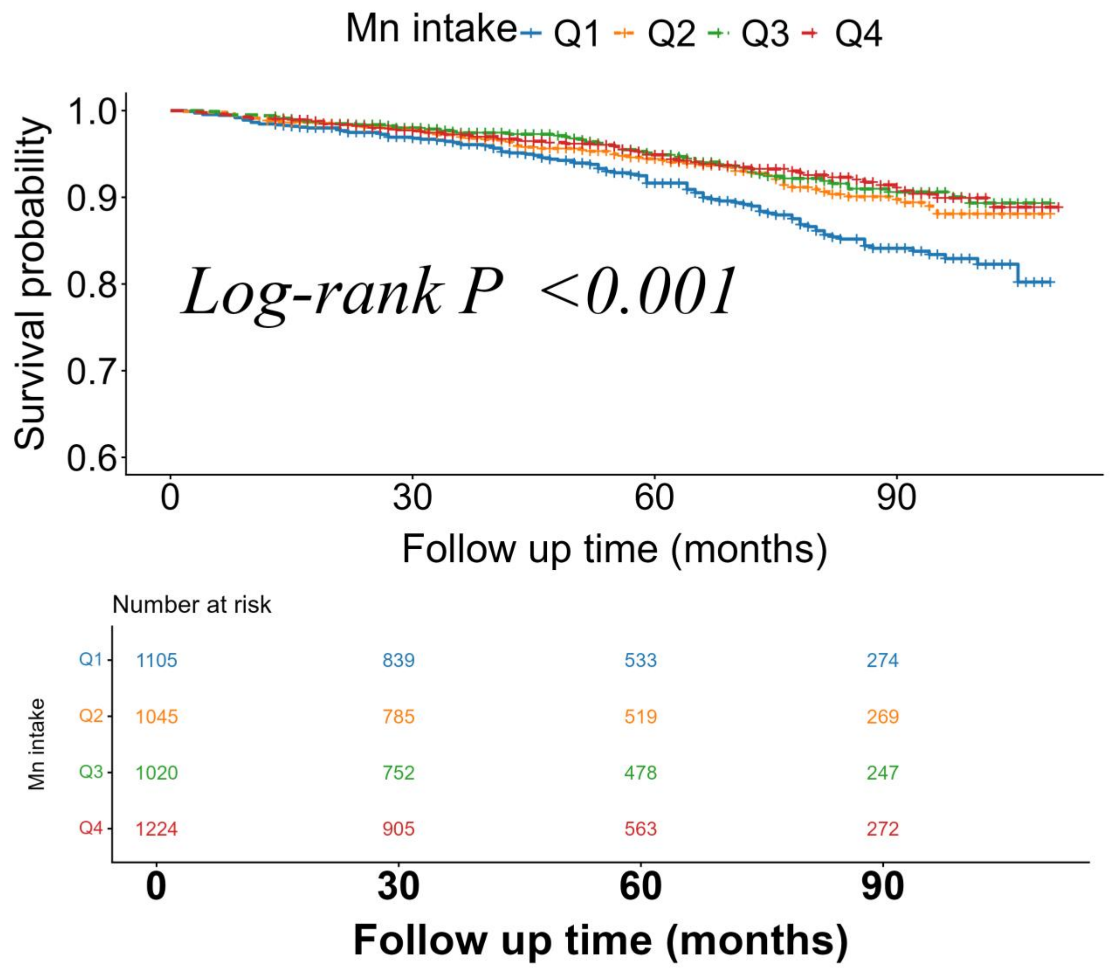

Mn intake
Q1
Q2
Q3
Q4
Survival probability
1.0
0.9
0.8
0.7
0.6
Log-rank P <0.001
0
30
60
90
Follow up time (months)
Number at risk
Mn intake
Q1 1105 839 533 274
Q2 1045 785 519 269
Q3 1020 752 478 247
Q4 1224 905 563 272
0
30
60
90
Follow up time (months)
