# Supplementary material for: Heavy metal exposure and risk of all-cause and cardiovascular mortality in population with cardiovascular-kidney-metabolic syndrome stage 0–3: a cohort study
Source: Environ Health Prev Med. 2026 Jul 3;31:45. doi: 10.1265/ehpm.26-00065 (PMC13366184; doi:10.1265/ehpm.26-00065)
Supplement: Supplementary file 3 — Additional file 3: Supplementary Table 1. Pairwise interaction analyses of blood metals in relation to mortality. [file ehpm-31-045-s003.pdf]

Online Resource 3.

Supplementary Table 1. Pairwise interaction analyses of blood metals in relation to mortality

Article title: Heavy metal exposure and risk of all-cause and cardiovascular mortality in population with cardiovascular-kidney-metabolic syndrome stage 0–3: a cohort study

Author information

Yiyang Liu1 · Fujian Li2 · Ying Huang1 · Jiansheng Cai1, 3· You Li1

1School of Public Health, Guilin Medical University, Guilin 541199, China
2Guangxi Hospital Division of the First Affiliated Hospital, Sun Yat-sen University, Nanning 530021, China

3Sub-Center of Key Laboratory of Environmental Pollution and Integrative Omics (Education Department of Guangxi Zhuang Autonomous Region), Lingshan Hospital of Guilin Medical University, Lingshan 535400, PR China

Yiyang Liu and Fujian Li equally contributed to this work.

✉ Corresponding authors:

You Li (liyou121300@163.com)
Jiansheng Cai (15007714226@163.com)

Description

Supplementary analyses of pairwise interactions among blood metals in relation to all-cause and cardiovascular mortality are shown in Online Resource 3 among adults with stage 0–3 cardiovascular–kidney–metabolic syndrome.

Multiplicative interactions were examined by including cross-product terms for each pair of metals in the fully adjusted survey-weighted Cox models. Blood concentrations of Pb, Cd, Hg, Se, and Mn were natural log-transformed and standardized before creating the interaction terms. For each interaction model, the two corresponding metal main effects and their cross-product term were included simultaneously.

Main findings

No statistically significant multiplicative interactions were observed between any pair of metals in relation to all-cause mortality or cardiovascular mortality. Specifically, the Pb × Cd interaction was not associated with all-cause mortality or cardiovascular mortality. For

all-cause mortality, the HR for the Pb × Cd interaction term was 0.978, with a 95% CI of 0.850–1.124 and a P for interaction of 0.7447. For cardiovascular mortality, the corresponding HR was 1.228, with a 95% CI of 0.940–1.605 and a P for interaction of 0.1473. After false discovery rate correction, none of the pairwise interaction terms remained statistically significant.

All models were adjusted for age, sex, educational attainment, race/ethnicity, alcohol consumption, physical activity, smoking status, and poverty income ratio. Survey weights, strata, and primary sampling units were incorporated in all analyses.

Supplementary Table 1. Pairwise interaction analyses of blood metals in relation to mortality

Outcome	Interaction	Beta	SE	HR	95% CI	P for interaction	FDR-adjusted P
All-cause mortality	Pb × Cd	-0.0227	0.0712	0.978	0.850–1.124	0.7447	0.9749
	Pb × Hg	-0.0130	0.0716	0.987	0.858–1.136	0.8628	0.9749
	Pb × Se	-0.0395	0.0704	0.961	0.837–1.104	0.6443	0.9749
	Pb × Mn	-0.0756	0.0751	0.927	0.800–1.074	0.4896	0.9749
	Cd × Hg	0.1107	0.0712	1.117	0.972–1.284	0.2336	0.9749
	Cd × Se	-0.0029	0.0630	0.997	0.881–1.128	0.9749	0.9749
	Cd × Mn	-0.0379	0.0750	0.963	0.831–1.115	0.6668	0.9749
	Hg × Se	-0.1181	0.0628	0.889	0.786–1.005	0.1029	0.9749
	Hg × Mn	-0.0139	0.0749	0.986	0.852–1.142	0.8825	0.9749
	Se × Mn	0.0541	0.0653	1.056	0.929–1.200	0.5134	0.9749

Cardiovascular mortality	Pb × Cd	0.2055	0.1365	1.228	0.940–1.605	0.1473	0.4910
	Pb × Hg	0.2188	0.1288	1.245	0.967–1.602	0.0599	0.4588
	Pb × Se	0.0455	0.1527	1.047	0.776–1.412	0.7401	0.9634
	Pb × Mn	0.2244	0.1552	1.252	0.923–1.697	0.0918	0.4588
	Cd × Hg	0.1765	0.1474	1.193	0.894–1.593	0.5373	0.9634
	Cd × Se	0.0086	0.1413	1.009	0.765–1.331	0.9572	0.9634
	Cd × Mn	0.0617	0.1600	1.064	0.777–1.455	0.7330	0.9634
	Hg × Se	-0.0331	0.1252	0.967	0.757–1.236	0.8208	0.9634
	Hg × Mn	0.1885	0.1363	1.207	0.924–1.577	0.2064	0.5160
	Se × Mn	-0.0065	0.1356	0.993	0.762–1.296		

Abbreviations: Pb, lead; Cd, cadmium; Hg, mercury; Se, selenium; Mn, manganese; HR, hazard ratio; CI, confidence interval; FDR, false discovery rate.

Note: Blood metal concentrations were natural log-transformed and standardized before creating pairwise interaction terms. Each model included the two corresponding metal main effects, their interaction term, and was adjusted for age, sex, educational attainment, race/ethnicity, alcohol consumption, physical activity, smoking status, and poverty income ratio. Survey weights, strata, and primary sampling units were incorporated. FDR-adjusted P values were calculated using the Benjamini-Hochberg method within each outcome.
